# Chicago Medical Society

**Published:** 1850-05

**Authors:** 


### ARTICLE V.

### CHICAGO MEDICAL SOCIETY.

A meeting of Physicians of Chicago was held at Drs. Boone and M'Vickar's office on the 15th ultimo. After considerable discussion a Society was organized, and a Committee appointed to draft a Constitution for its government.

On the 19th ult. the Society met again, and after adopting a Constitution, elected Dr. L. D. Boone, President; E. McArthur, Vice-President; B. McVickar, Secretary, and Drs. Evans and Boone Delegates to the American Medical Association.

Much good feeling was manifested, and the Society promises a career of usefulness.

The stated Meetings are to be held on the first Monday in each month.

